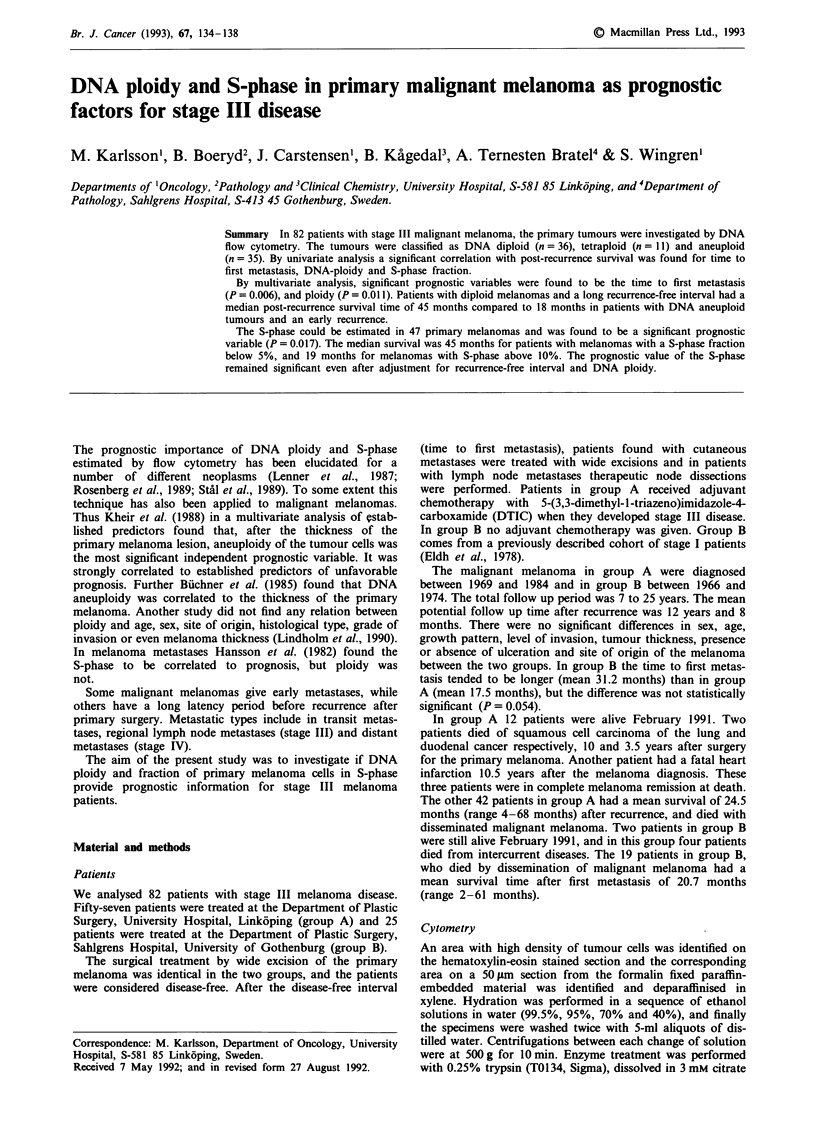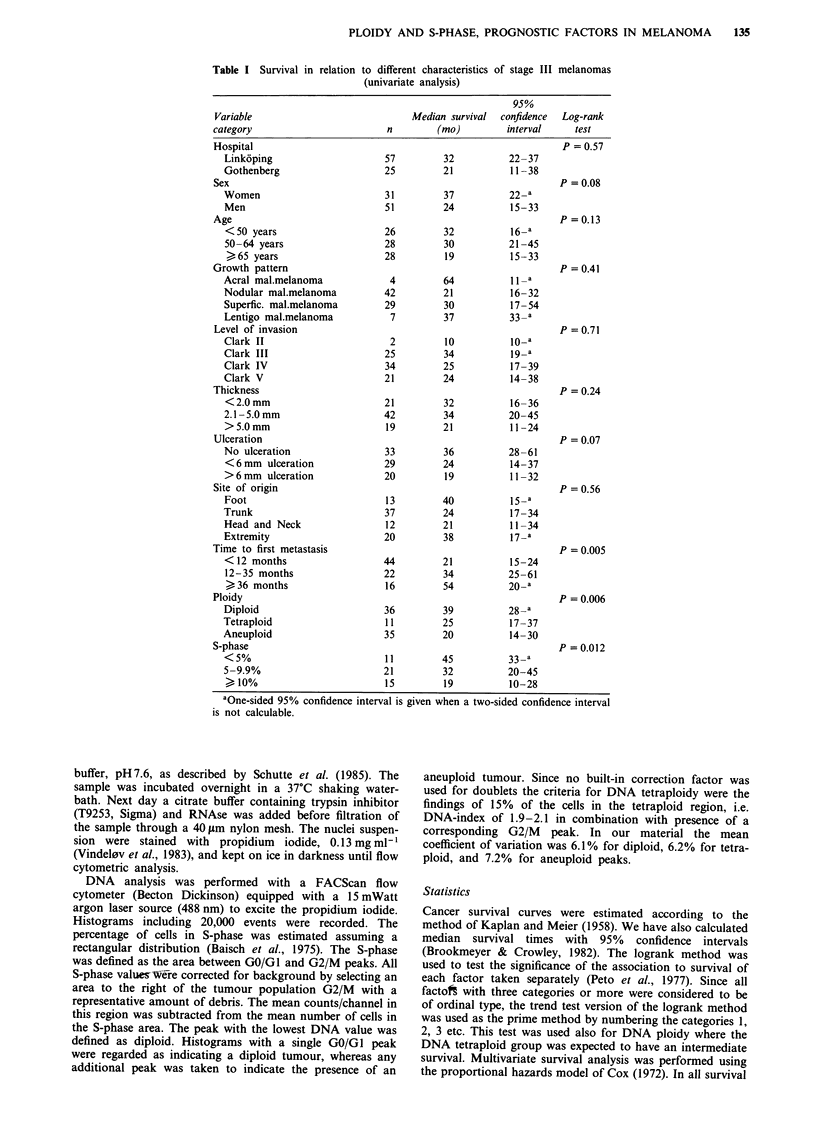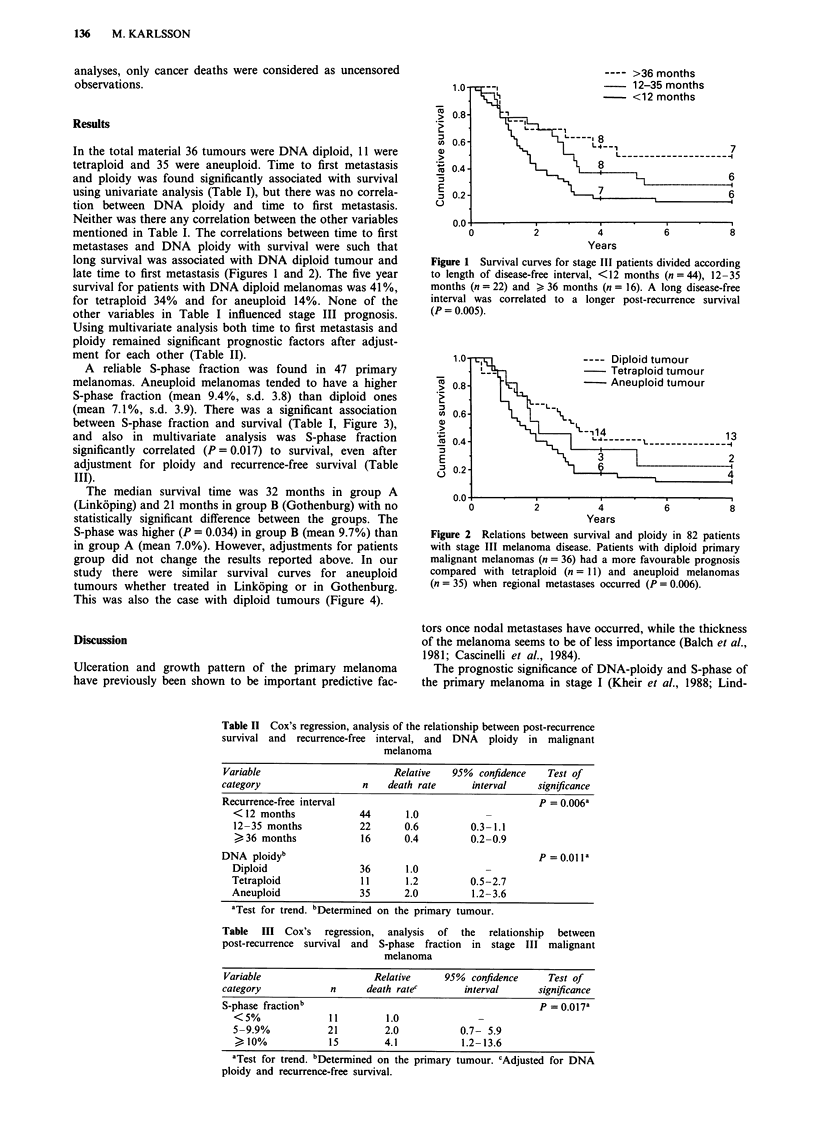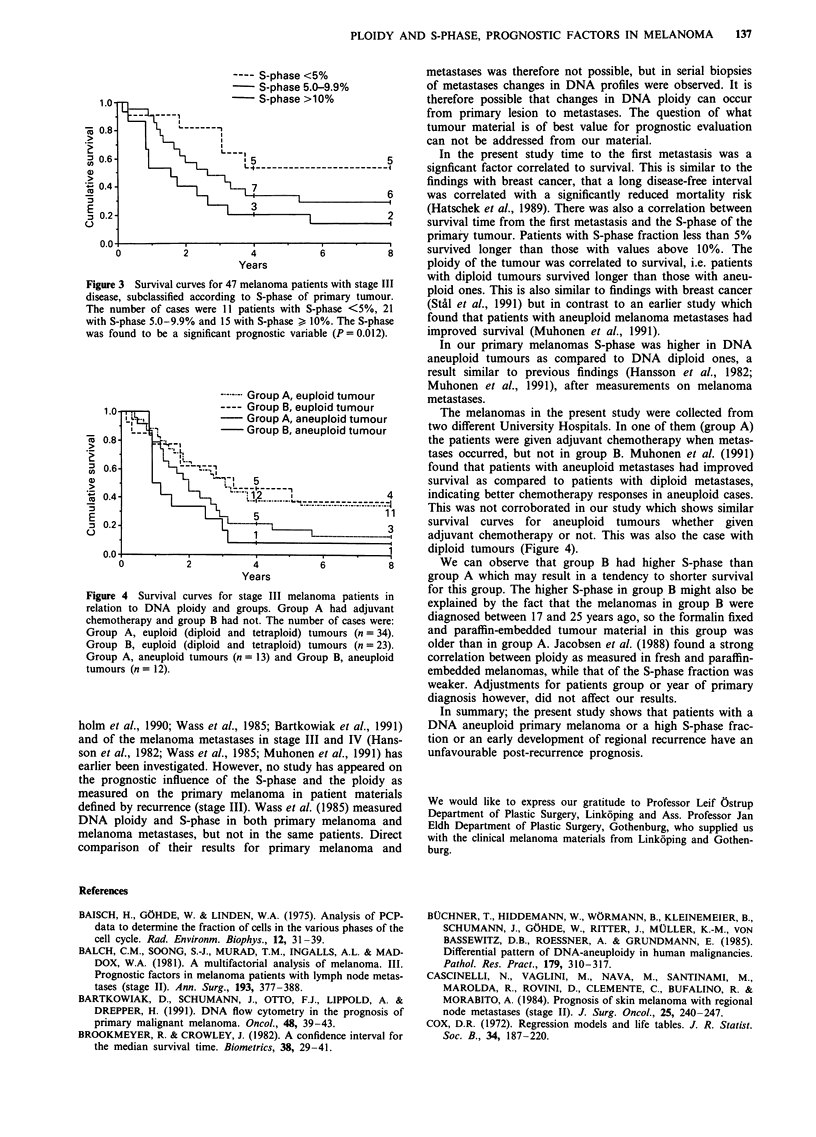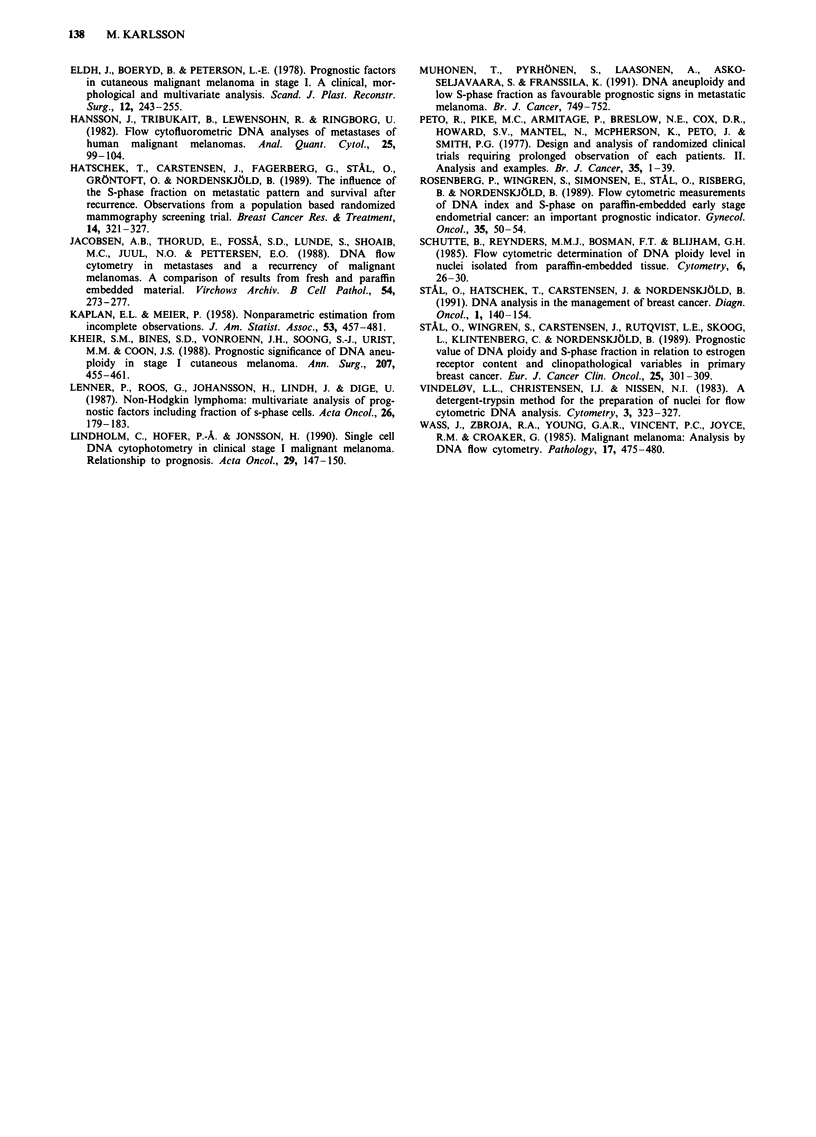# DNA ploidy and S-phase in primary malignant melanoma as prognostic factors for stage III disease.

**DOI:** 10.1038/bjc.1993.23

**Published:** 1993-01

**Authors:** M. Karlsson, B. Boeryd, J. Carstensen, B. Kågedal, A. T. Bratel, S. Wingren

**Affiliations:** Department of Oncology, University Hospital, Linköping, Sweden.

## Abstract

In 82 patients with stage III malignant melanoma, the primary tumours were investigated by DNA flow cytometry. The tumours were classified as DNA diploid (n = 36), tetraploid (n = 11) and aneuploid (n = 35). By univariate analysis a significant correlation with post-recurrence survival was found for time to first metastasis, DNA-ploidy and S-phase fraction. By multivariate analysis, significant prognostic variables were found to be the time to first metastasis (P = 0.006), and ploidy (P = 0.011). Patients with diploid melanomas and a long recurrence-free interval had a median post-recurrence survival time of 45 months compared to 18 months in patients with DNA aneuploid tumours and an early recurrence. The S-phase could be estimated in 47 primary melanomas and was found to be a significant prognostic variable (P = 0.017). The median survival was 45 months for patients with melanomas with a S-phase fraction below 5%, and 19 months for melanomas with S-phase above 10%. The prognostic value of the S-phase remained significant even after adjustment for recurrence-free interval and DNA ploidy.


Br. J. Cancer (1993), 67, 134–138 © Macmillan Press Ltd., 1993

### DNA ploidy and S-phase in primary malignant melanoma as prognostic factors for stage III disease

M. Karlsson[1], B. Boeryd[2], J. Carstensen[1], B. Kågedal[3], A. Ternesten Bratel[4] & S. Wingren[1]

*Departments of [1]Oncology, [2]Pathology and [3]Clinical Chemistry, University Hospital, S-581 85 Linköping, and [4]Department of Pathology, Sahlgrens Hospital, S-413 45 Gothenburg, Sweden.*

**Summary** In 82 patients with stage III malignant melanoma, the primary tumours were investigated by DNA flow cytometry. The tumours were classified as DNA diploid ($n = 36$), tetraploid ($n = 11$) and aneuploid ($n = 35$). By univariate analysis a significant correlation with post-recurrence survival was found for time to first metastasis, DNA-ploidy and S-phase fraction.

By multivariate analysis, significant prognostic variables were found to be the time to first metastasis ($P = 0.006$), and ploidy ($P = 0.011$). Patients with diploid melanomas and a long recurrence-free interval had a median post-recurrence survival time of 45 months compared to 18 months in patients with DNA aneuploid tumours and an early recurrence.

The S-phase could be estimated in 47 primary melanomas and was found to be a significant prognostic variable ($P = 0.017$). The median survival was 45 months for patients with melanomas with a S-phase fraction below 5%, and 19 months for melanomas with S-phase above 10%. The prognostic value of the S-phase remained significant even after adjustment for recurrence-free interval and DNA ploidy.

The prognostic importance of DNA ploidy and S-phase estimated by flow cytometry has been elucidated for a number of different neoplasms (Lenner *et al.*, 1987; Rosenberg *et al.*, 1989; Stål *et al.*, 1989). To some extent this technique has also been applied to malignant melanomas. Thus Kheir *et al.* (1988) in a multivariate analysis of established predictors found that, after the thickness of the primary melanoma lesion, aneuploidy of the tumour cells was the most significant independent prognostic variable. It was strongly correlated to established predictors of unfavorable prognosis. Further Büchner *et al.* (1985) found that DNA aneuploidy was correlated to the thickness of the primary melanoma. Another study did not find any relation between ploidy and age, sex, site of origin, histological type, grade of invasion or even melanoma thickness (Lindholm *et al.*, 1990). In melanoma metastases Hansson *et al.* (1982) found the S-phase to be correlated to prognosis, but ploidy was not.

Some malignant melanomas give early metastases, while others have a long latency period before recurrence after primary surgery. Metastatic types include in transit metastases, regional lymph node metastases (stage III) and distant metastases (stage IV).

The aim of the present study was to investigate if DNA ploidy and fraction of primary melanoma cells in S-phase provide prognostic information for stage III melanoma patients.

### Material and methods

### *Patients*

We analysed 82 patients with stage III melanoma disease. Fifty-seven patients were treated at the Department of Plastic Surgery, University Hospital, Linköping (group A) and 25 patients were treated at the Department of Plastic Surgery, Sahlgrens Hospital, University of Gothenburg (group B).

The surgical treatment by wide excision of the primary melanoma was identical in the two groups, and the patients were considered disease-free. After the disease-free interval (time to first metastasis), patients found with cutaneous metastases were treated with wide excisions and in patients with lymph node metastases therapeutic node dissections were performed. Patients in group A received adjuvant chemotherapy with 5-(3,3-dimethyl-1-triazeno)imidazole-4-carboxamide (DTIC) when they developed stage III disease. In group B no adjuvant chemotherapy was given. Group B comes from a previously described cohort of stage I patients (Eldh *et al.*, 1978).

The malignant melanoma in group A were diagnosed between 1969 and 1984 and in group B between 1966 and 1974. The total follow up period was 7 to 25 years. The mean potential follow up time after recurrence was 12 years and 8 months. There were no significant differences in sex, age, growth pattern, level of invasion, tumour thickness, presence or absence of ulceration and site of origin of the melanoma between the two groups. In group B the time to first metastasis tended to be longer (mean 31.2 months) than in group A (mean 17.5 months), but the difference was not statistically significant ($P = 0.054$).

In group A 12 patients were alive February 1991. Two patients died of squamous cell carcinoma of the lung and duodenal cancer respectively, 10 and 3.5 years after surgery for the primary melanoma. Another patient had a fatal heart infarction 10.5 years after the melanoma diagnosis. These three patients were in complete melanoma remission at death. The other 42 patients in group A had a mean survival of 24.5 months (range 4–68 months) after recurrence, and died with disseminated malignant melanoma. Two patients in group B were still alive February 1991, and in this group four patients died from intercurrent diseases. The 19 patients in group B, who died by dissemination of malignant melanoma had a mean survival time after first metastasis of 20.7 months (range 2–61 months).

### *Cytometry*

An area with high density of tumour cells was identified on the hematoxylin-eosin stained section and the corresponding area on a 50 μm section from the formalin fixed paraffin-embedded material was identified and deparaffinised in xylene. Hydration was performed in a sequence of ethanol solutions in water (99.5%, 95%, 70% and 40%), and finally the specimens were washed twice with 5-ml aliquots of distilled water. Centrifugations between each change of solution were at 500 g for 10 min. Enzyme treatment was performed with 0.25% trypsin (T0134, Sigma), dissolved in 3 mM citrate

Correspondence: M. Karlsson, Department of Oncology, University Hospital, S-581 85 Linköping, Sweden.
Received 7 May 1992; and in revised form 27 August 1992.

**Table I** Survival in relation to different characteristics of stage III melanomas (univariate analysis)

| *Variable category* | *n* | *Median survival (mo)* | *95% confidence interval* | *Log-rank test* |
|---|---|---|---|---|
| Hospital | | | | $P = 0.57$ |
| Linköping | 57 | 32 | 22–37 | |
| Gothenberg | 25 | 21 | 11–38 | |
| Sex | | | | $P = 0.08$ |
| Women | 31 | 37 | 22–[a] | |
| Men | 51 | 24 | 15–33 | |
| Age | | | | $P = 0.13$ |
| <50 years | 26 | 32 | 16–[a] | |
| 50–64 years | 28 | 30 | 21–45 | |
| ⩾65 years | 28 | 19 | 15–33 | |
| Growth pattern | | | | $P = 0.41$ |
| Acral mal.melanoma | 4 | 64 | 11–[a] | |
| Nodular mal.melanoma | 42 | 21 | 16–32 | |
| Superfic. mal.melanoma | 29 | 30 | 17–54 | |
| Lentigo mal.melanoma | 7 | 37 | 33–[a] | |
| Level of invasion | | | | $P = 0.71$ |
| Clark II | 2 | 10 | 10–[a] | |
| Clark III | 25 | 34 | 19–[a] | |
| Clark IV | 34 | 25 | 17–39 | |
| Clark V | 21 | 24 | 14–38 | |
| Thickness | | | | $P = 0.24$ |
| <2.0 mm | 21 | 32 | 16–36 | |
| 2.1–5.0 mm | 42 | 34 | 20–45 | |
| >5.0 mm | 19 | 21 | 11–24 | |
| Ulceration | | | | $P = 0.07$ |
| No ulceration | 33 | 36 | 28–61 | |
| <6 mm ulceration | 29 | 24 | 14–37 | |
| >6 mm ulceration | 20 | 19 | 11–32 | |
| Site of origin | | | | $P = 0.56$ |
| Foot | 13 | 40 | 15–[a] | |
| Trunk | 37 | 24 | 17–34 | |
| Head and Neck | 12 | 21 | 11–34 | |
| Extremity | 20 | 38 | 17–[a] | |
| Time to first metastasis | | | | $P = 0.005$ |
| <12 months | 44 | 21 | 15–24 | |
| 12–35 months | 22 | 34 | 25–61 | |
| ⩾36 months | 16 | 54 | 20–[a] | |
| Ploidy | | | | $P = 0.006$ |
| Diploid | 36 | 39 | 28–[a] | |
| Tetraploid | 11 | 25 | 17–37 | |
| Aneuploid | 35 | 20 | 14–30 | |
| S-phase | | | | $P = 0.012$ |
| <5% | 11 | 45 | 33–[a] | |
| 5–9.9% | 21 | 32 | 20–45 | |
| ⩾10% | 15 | 19 | 10–28 | |

[a]One-sided 95% confidence interval is given when a two-sided confidence interval is not calculable.

buffer, pH7.6, as described by Schutte *et al.* (1985). The sample was incubated overnight in a 37°C shaking water-bath. Next day a citrate buffer containing trypsin inhibitor (T9253, Sigma) and RNAse was added before filtration of the sample through a 40 μm nylon mesh. The nuclei suspension were stained with propidium iodide, 0.13 mg ml$^{-1}$ (Vindeløv *et al.*, 1983), and kept on ice in darkness until flow cytometric analysis.

DNA analysis was performed with a FACScan flow cytometer (Becton Dickinson) equipped with a 15 mWatt argon laser source (488 nm) to excite the propidium iodide. Histograms including 20,000 events were recorded. The percentage of cells in S-phase was estimated assuming a rectangular distribution (Baisch *et al.*, 1975). The S-phase was defined as the area between G0/G1 and G2/M peaks. All S-phase values were corrected for background by selecting an area to the right of the tumour population G2/M with a representative amount of debris. The mean counts/channel in this region was subtracted from the mean number of cells in the S-phase area. The peak with the lowest DNA value was defined as diploid. Histograms with a single G0/G1 peak were regarded as indicating a diploid tumour, whereas any additional peak was taken to indicate the presence of an aneuploid tumour. Since no built-in correction factor was used for doublets the criteria for DNA tetraploidy were the findings of 15% of the cells in the tetraploid region, i.e. DNA-index of 1.9–2.1 in combination with presence of a corresponding G2/M peak. In our material the mean coefficient of variation was 6.1% for diploid, 6.2% for tetraploid, and 7.2% for aneuploid peaks.

### *Statistics*

Cancer survival curves were estimated according to the method of Kaplan and Meier (1958). We have also calculated median survival times with 95% confidence intervals (Brookmeyer & Crowley, 1982). The logrank method was used to test the significance of the association to survival of each factor taken separately (Peto *et al.*, 1977). Since all factors with three categories or more were considered to be of ordinal type, the trend test version of the logrank method was used as the prime method by numbering the categories 1, 2, 3 etc. This test was used also for DNA ploidy where the DNA tetraploid group was expected to have an intermediate survival. Multivariate survival analysis was performed using the proportional hazards model of Cox (1972). In all survival analyses, only cancer deaths were considered as uncensored observations.

### Results

In the total material 36 tumours were DNA diploid, 11 were tetraploid and 35 were aneuploid. Time to first metastasis and ploidy was found significantly associated with survival using univariate analysis (Table I), but there was no correlation between DNA ploidy and time to first metastasis. Neither was there any correlation between the other variables mentioned in Table I. The correlations between time to first metastases and DNA ploidy with survival were such that long survival was associated with DNA diploid tumour and late time to first metastasis (Figures 1 and 2). The five year survival for patients with DNA diploid melanomas was 41%, for tetraploid 34% and for aneuploid 14%. None of the other variables in Table I influenced stage III prognosis. Using multivariate analysis both time to first metastasis and ploidy remained significant prognostic factors after adjustment for each other (Table II).

A reliable S-phase fraction was found in 47 primary melanomas. Aneuploid melanomas tended to have a higher S-phase fraction (mean 9.4%, s.d. 3.8) than diploid ones (mean 7.1%, s.d. 3.9). There was a significant association between S-phase fraction and survival (Table I, Figure 3), and also in multivariate analysis was S-phase fraction significantly correlated ($P = 0.017$) to survival, even after adjustment for ploidy and recurrence-free survival (Table III).

The median survival time was 32 months in group A (Linköping) and 21 months in group B (Gothenburg) with no statistically significant difference between the groups. The S-phase was higher ($P = 0.034$) in group B (mean 9.7%) than in group A (mean 7.0%). However, adjustments for patients group did not change the results reported above. In our study there were similar survival curves for aneuploid tumours whether treated in Linköping or in Gothenburg. This was also the case with diploid tumours (Figure 4).

**Figure 1** Survival curves for stage III patients divided according to length of disease-free interval, <12 months ($n = 44$), 12–35 months ($n = 22$) and ⩾36 months ($n = 16$). A long disease-free interval was correlated to a longer post-recurrence survival ($P = 0.005$).

**Figure 2** Relations between survival and ploidy in 82 patients with stage III melanoma disease. Patients with diploid primary malignant melanomas ($n = 36$) had a more favourable prognosis compared with tetraploid ($n = 11$) and aneuploid melanomas ($n = 35$) when regional metastases occurred ($P = 0.006$).

### Discussion

Ulceration and growth pattern of the primary melanoma have previously been shown to be important predictive factors once nodal metastases have occurred, while the thickness of the melanoma seems to be of less importance (Balch *et al.*, 1981; Cascinelli *et al.*, 1984).

The prognostic significance of DNA-ploidy and S-phase of the primary melanoma in stage I (Kheir *et al.*, 1988; Lind-

**Table II** Cox's regression, analysis of the relationship between post-recurrence survival and recurrence-free interval, and DNA ploidy in malignant melanoma

| *Variable category* | *n* | *Relative death rate* | *95% confidence interval* | *Test of significance* |
|---|---|---|---|---|
| Recurrence-free interval | | | | $P = 0.006$[a] |
| <12 months | 44 | 1.0 | – | |
| 12–35 months | 22 | 0.6 | 0.3–1.1 | |
| ⩾36 months | 16 | 0.4 | 0.2–0.9 | |
| DNA ploidy[b] | | | | $P = 0.011$[a] |
| Diploid | 36 | 1.0 | – | |
| Tetraploid | 11 | 1.2 | 0.5–2.7 | |
| Aneuploid | 35 | 2.0 | 1.2–3.6 | |

[a]Test for trend. [b]Determined on the primary tumour.

**Table III** Cox's regression, analysis of the relationship between post-recurrence survival and S-phase fraction in stage III malignant melanoma

| *Variable category* | *n* | *Relative death rate*[c] | *95% confidence interval* | *Test of significance* |
|---|---|---|---|---|
| S-phase fraction[b] | | | | $P = 0.017$[a] |
| <5% | 11 | 1.0 | – | |
| 5–9.9% | 21 | 2.0 | 0.7– 5.9 | |
| ⩾10% | 15 | 4.1 | 1.2–13.6 | |

[a]Test for trend. [b]Determined on the primary tumour. [c]Adjusted for DNA ploidy and recurrence-free survival.

**Figure 3** Survival curves for 47 melanoma patients with stage III disease, subclassified according to S-phase of primary tumour. The number of cases were 11 patients with S-phase <5%, 21 with S-phase 5.0–9.9% and 15 with S-phase ⩾ 10%. The S-phase was found to be a significant prognostic variable ($P = 0.012$).

**Figure 4** Survival curves for stage III melanoma patients in relation to DNA ploidy and groups. Group A had adjuvant chemotherapy and group B had not. The number of cases were: Group A, euploid (diploid and tetraploid) tumours ($n = 34$). Group B, euploid (diploid and tetraploid) tumours ($n = 23$). Group A, aneuploid tumours ($n = 13$) and Group B, aneuploid tumours ($n = 12$).

holm *et al.*, 1990; Wass *et al.*, 1985; Bartkowiak *et al.*, 1991) and of the melanoma metastases in stage III and IV (Hansson *et al.*, 1982; Wass *et al.*, 1985; Muhonen *et al.*, 1991) has earlier been investigated. However, no study has appeared on the prognostic influence of the S-phase and the ploidy as measured on the primary melanoma in patient materials defined by recurrence (stage III). Wass *et al.* (1985) measured DNA ploidy and S-phase in both primary melanoma and melanoma metastases, but not in the same patients. Direct comparison of their results for primary melanoma and metastases was therefore not possible, but in serial biopsies of metastases changes in DNA profiles were observed. It is therefore possible that changes in DNA ploidy can occur from primary lesion to metastases. The question of what tumour material is of best value for prognostic evaluation can not be addressed from our material.

In the present study time to the first metastasis was a signficant factor correlated to survival. This is similar to the findings with breast cancer, that a long disease-free interval was correlated with a significantly reduced mortality risk (Hatschek *et al.*, 1989). There was also a correlation between survival time from the first metastasis and the S-phase of the primary tumour. Patients with S-phase fraction less than 5% survived longer than those with values above 10%. The ploidy of the tumour was correlated to survival, i.e. patients with diploid tumours survived longer than those with aneuploid ones. This is also similar to findings with breast cancer (Stål *et al.*, 1991) but in contrast to an earlier study which found that patients with aneuploid melanoma metastases had improved survival (Muhonen *et al.*, 1991).

In our primary melanomas S-phase was higher in DNA aneuploid tumours as compared to DNA diploid ones, a result similar to previous findings (Hansson *et al.*, 1982; Muhonen *et al.*, 1991), after measurements on melanoma metastases.

The melanomas in the present study were collected from two different University Hospitals. In one of them (group A) the patients were given adjuvant chemotherapy when metastases occurred, but not in group B. Muhonen *et al.* (1991) found that patients with aneuploid metastases had improved survival as compared to patients with diploid metastases, indicating better chemotherapy responses in aneuploid cases. This was not corroborated in our study which shows similar survival curves for aneuploid tumours whether given adjuvant chemotherapy or not. This was also the case with diploid tumours (Figure 4).

We can observe that group B had higher S-phase than group A which may result in a tendency to shorter survival for this group. The higher S-phase in group B might also be explained by the fact that the melanomas in group B were diagnosed between 17 and 25 years ago, so the formalin fixed and paraffin-embedded tumour material in this group was older than in group A. Jacobsen *et al.* (1988) found a strong correlation between ploidy as measured in fresh and paraffin-embedded melanomas, while that of the S-phase fraction was weaker. Adjustments for patients group or year of primary diagnosis however, did not affect our results.

In summary; the present study shows that patients with a DNA aneuploid primary melanoma or a high S-phase fraction or an early development of regional recurrence have an unfavourable post-recurrence prognosis.

We would like to express our gratitude to Professor Leif Östrup Department of Plastic Surgery, Linköping and Ass. Professor Jan Eldh Department of Plastic Surgery, Gothenburg, who supplied us with the clinical melanoma materials from Linköping and Gothenburg.

### References

BAISCH, H., GÖHDE, W. & LINDEN, W.A. (1975). Analysis of PCP-data to determine the fraction of cells in the various phases of the cell cycle. *Rad. Environm. Biophys.*, **12**, 31–39.

BALCH, C.M., SOONG, S.-J., MURAD, T.M., INGALLS, A.L. & MADDOX, W.A. (1981). A multifactorial analysis of melanoma. III. Prognostic factors in melanoma patients with lymph node metastases (stage II). *Ann. Surg.*, **193**, 377–388.

BARTKOWIAK, D., SCHUMANN, J., OTTO, F.J., LIPPOLD, A. & DREPPER, H. (1991). DNA flow cytometry in the prognosis of primary malignant melanoma. *Oncol.*, **48**, 39–43.

BROOKMEYER, R. & CROWLEY, J. (1982). A confidence interval for the median survival time. *Biometrics*, **38**, 29–41.

BÜCHNER, T., HIDDEMANN, W., WÖRMANN, B., KLEINEMEIER, B., SCHUMANN, J., GÖHDE, W., RITTER, J., MÜLLER, K.-M., VON BASSEWITZ, D.B., ROESSNER, A. & GRUNDMANN, E. (1985). Differential pattern of DNA-aneuploidy in human malignancies. *Pathol. Res. Pract.*, **179**, 310–317.

CASCINELLI, N., VAGLINI, M., NAVA, M., SANTINAMI, M., MAROLDA, R., ROVINI, D., CLEMENTE, C., BUFALINO, R. & MORABITO, A. (1984). Prognosis of skin melanoma with regional node metastases (stage II). *J. Surg. Oncol.*, **25**, 240–247.

COX, D.R. (1972). Regression models and life tables. *J. R. Statist. Soc. B.*, **34**, 187–220.

ELDH, J., BOERYD, B. & PETERSON, L.-E. (1978). Prognostic factors in cutaneous malignant melanoma in stage I. A clinical, morphological and multivariate analysis. *Scand. J. Plast. Reconstr. Surg.*, **12**, 243–255.

HANSSON, J., TRIBUKAIT, B., LEWENSOHN, R. & RINGBORG, U. (1982). Flow cytofluorometric DNA analyses of metastases of human malignant melanomas. *Anal. Quant. Cytol.*, **25**, 99–104.

HATSCHEK, T., CARSTENSEN, J., FAGERBERG, G., STÅL, O., GRÖNTOFT, O. & NORDENSKJÖLD, B. (1989). The influence of the S-phase fraction on metastatic pattern and survival after recurrence. Observations from a population based randomized mammography screening trial. *Breast Cancer Res. & Treatment*, **14**, 321–327.

JACOBSEN, A.B., THORUD, E., FOSSÅ, S.D., LUNDE, S., SHOAIB, M.C., JUUL, N.O. & PETTERSEN, E.O. (1988). DNA flow cytometry in metastases and a recurrency of malignant melanomas. A comparison of results from fresh and paraffin embedded material. *Virchows Archiv. B Cell Pathol.*, **54**, 273–277.

KAPLAN, E.L. & MEIER, P. (1958). Nonparametric estimation from incomplete observations. *J. Am. Statist. Assoc.*, **53**, 457–481.

KHEIR, S.M., BINES, S.D., VONROENN, J.H., SOONG, S.-J., URIST, M.M. & COON, J.S. (1988). Prognostic significance of DNA aneuploidy in stage I cutaneous melanoma. *Ann. Surg.*, **207**, 455–461.

LENNER, P., ROOS, G., JOHANSSON, H., LINDH, J. & DIGE, U. (1987). Non-Hodgkin lymphoma: multivariate analysis of prognostic factors including fraction of s-phase cells. *Acta Oncol.*, **26**, 179–183.

LINDHOLM, C., HOFER, P.-Å. & JONSSON, H. (1990). Single cell DNA cytophotometry in clinical stage I malignant melanoma. Relationship to prognosis. *Acta Oncol.*, **29**, 147–150.

MUHONEN, T., PYRHÖNEN, S., LAASONEN, A., ASKO-SELJAVAARA, S. & FRANSSILA, K. (1991). DNA aneuploidy and low S-phase fraction as favourable prognostic signs in metastatic melanoma. *Br. J. Cancer*, 749–752.

PETO, R., PIKE, M.C., ARMITAGE, P., BRESLOW, N.E., COX, D.R., HOWARD, S.V., MANTEL, N., MCPHERSON, K., PETO, J. & SMITH, P.G. (1977). Design and analysis of randomized clinical trials requiring prolonged observation of each patients. II. Analysis and examples. *Br. J. Cancer*, **35**, 1–39.

ROSENBERG, P., WINGREN, S., SIMONSEN, E., STÅL, O., RISBERG, B. & NORDENSKJÖLD, B. (1989). Flow cytometric measurements of DNA index and S-phase on paraffin-embedded early stage endometrial cancer: an important prognostic indicator. *Gynecol. Oncol.*, **35**, 50–54.

SCHUTTE, B., REYNDERS, M.M.J., BOSMAN, F.T. & BLIJHAM, G.H. (1985). Flow cytometric determination of DNA ploidy level in nuclei isolated from paraffin-embedded tissue. *Cytometry*, **6**, 26–30.

STÅL, O., HATSCHEK, T., CARSTENSEN, J. & NORDENSKJÖLD, B. (1991). DNA analysis in the management of breast cancer. *Diagn. Oncol.*, **1**, 140–154.

STÅL, O., WINGREN, S., CARSTENSEN, J., RUTQVIST, L.E., SKOOG, L., KLINTENBERG, C. & NORDENSKJÖLD, B. (1989). Prognostic value of DNA ploidy and S-phase fraction in relation to estrogen receptor content and clinopathological variables in primary breast cancer. *Eur. J. Cancer Clin. Oncol.*, **25**, 301–309.

VINDELØV, L.L., CHRISTENSEN, I.J. & NISSEN, N.I. (1983). A detergent-trypsin method for the preparation of nuclei for flow cytometric DNA analysis. *Cytometry*, **3**, 323–327.

WASS, J., ZBROJA, R.A., YOUNG, G.A.R., VINCENT, P.C., JOYCE, R.M. & CROAKER, G. (1985). Malignant melanoma: Analysis by DNA flow cytometry. *Pathology*, **17**, 475–480.